# Learners’ perceived advantages and social-affective dispositions toward online peer feedback in academic writing

**DOI:** 10.3389/fpsyg.2022.973478

**Published:** 2022-09-20

**Authors:** Meng Zhang, Qiaoling He, Jianxia Du, Fangtong Liu, Bosu Huang

**Affiliations:** ^1^School of English Studies, Sichuan International Studies University, Chongqing, China; ^2^College of General Education, Sichuan International Studies University, Chongqing, China; ^3^Department of English and German Studies, University of Rovira i Virgili, Tarragona, Spain; ^4^Facaulty of Education, University of Macau, Taipa, Macao SAR, China; ^5^School of Foreign Language Studies, Shanxi University, Taiyuan, China

**Keywords:** peer feedback, academic writing, EFL classroom practice, online feedback, social-affective disposition, technological features

## Abstract

Peer feedback is widely acknowledged for its advantages and benefits in improving students’ learning in writing classes. Although the integration of online platforms has been found to impact peer feedback, research on second language learners’ perceived advantages of social affective disposition to using multiple platforms for delivering peer feedback is limited. To address the aforementioned research gap, we conducted this 12-week action research to explore how 12 doctoral students at a university in Macau perceived their experience of using multiple online feedbacks in an academic writing course. To integrate the various advantages of different online platforms, we adopted three tools including Moodle, Rain Classroom, and WeChat for the delivery of peer feedback. The results demonstrated learners’ perceived advantages and disadvantages of online peer feedback and how the different online peer feedback can be combined to magnify their benefits for academic writing. It also revealed that the use of emojis, memes, and one-to-one conversation window on WeChat can foster students’ positive emotions. However, the ubiquitous connection by WeChat Moments increased their emotional load and undermined peer trust.

## Introduction

Peer feedback (PF), one of the most important teaching strategies, has been extensively used in educational fields such as second language (L2) learning and teaching. Previous studies have shown the beneficial role of PF in improving L2 learners’ writing efficacy (Loewen and Sato, 2018; Lee and Evans, 2019) and sustaining their learning interests (Lin and Chien, 2009; Yu and Lee, 2014, 2016).

As technology has permeated practically every area of L2 teaching and learning, online PF therefore has attracted extensive concerns from researchers and practitioners. Mediating technological tools for conducting PF evolved from MS word files, PDF editor, Learning Management System (LMS), social media, and specialized online feedback tools. The constant development and updating of technological tools revealed the necessity of integrating advantageous functions from different tools for feedback giving and receiving.

It has been recognized that using different technological tools influences feedback effects in different ways, since different technological platforms cast respective strengths on PF. However, little research has explored how the combination of different online tools affected L2 learners’ perceived pros and cons of PF. Moreover, different tools altogether created a unique affective atmosphere for students to give and receive feedback, from more formal and serious ones in LMS to more relaxed contexts in social media. Although previous research suggested that emotions students perceived from feedback strongly correlate with the acceptability of their feedback (G. Lin, 2018), to our knowledge, no study has ever addressed learners’ social affective dispositions toward the online feedback process.

In respect to the two research gaps as mentioned above, the present study introduced integrated online platforms consisting of LMS, social media, and real-time feedback tools for doctoral students to give and receive PF in an academic writing course. The current study examined the characteristics of the students’ perceived benefits and social-affective dispositions toward online PF *via* the integrated online platform in practice during a 12-week period of observation and data collection.

## Literature review

### Online peer feedback as a writing pedagogy

Peer feedback (PF), also referred to as “peer assessment,” “peer evaluation,” “peer review,” and “peer comments,” was a critical pedagogy making students aware of the strengths and weaknesses of their performance in their peers’ views (Topping and Ehly, 1998; Topping, 2009). In the peer feedback activity, learners replace the roles and responsibilities of a qualified teacher, tutor, or editor, in commenting on one another’s learning output. It is believed PF not only provides the audience perspective for feedback receivers to reexamine and improve their works, but also trains feedback givers’ reflective and critical thinking skills (Topping and Ehly, 1998; Liu and Carless, 2006). Over recent decades, PF has been regarded as an important building block in L2 writing (Yu and Lee, 2016; Hyland and Hyland(eds), 2019). The significance of PF as writing instruction has been supported by process writing theory (Hayes, 2012). Process writing theory emphasizes the process rather than the result of writing, viewing the writing process as a dynamic, nonlinear, and recursive process of meaning-making and knowledge transmitting. The role of PF is to provide information about the authors’ performance at the different stages of the writing process and help authors to improve their writing content, develop and practice writing skills (Yusof et al., 2012). Previous research (e.g., Guardado and Shi, 2007; Hamid et al., 2014; Yu and Lee, 2014; Nguyen et al., 2017; Yu and Hu, 2017; Lee and Evans, 2019) reported that PF have brought a variety of benefits to L2 learners, including improvement in language quality, reader awareness, autonomous learning skills, self-regulated learning skills, and language learning motivation.

Furthermore, the introduction of computer-mediated tools along with the rapid development of information and communication technology led to the emergence of various computer-mediated PF and accelerated the development of computer-mediated PF [for a review, see Chen (2016)]. Computer-mediated PF has developed from text based track-changes with MS Word (Liu and Sadler, 2003; AbuSeileek and Abualsha’r, 2014), audio and video-based (Rassaei, 2019), screencast (Cunningham, 2019), LMS such as Moodle or Blackboard (Fernando, 2020), to the incorporation of online social media with synchronous chat (Chang, 2012) such as WhatsApp and bulletin-board posting (Guardado and Shi, 2007). Over recent years, online PF has become a popular research topic due to the springing up of Web 2.0 tools (e.g., wiki, blog, social networking sites, media sharing sites, cloud computing, etc.) (Mei et al., 2018). The functions of various online tools have been widely recognized in previous studies. For example, research showed that online PF can extend students’ learning time outside classroom (Cheng, 2009), benefit their overall test scores (AbuSeileek and Abualsha’r, 2014), help them achieve better writing performance in their revised drafts (Ciftci and Kocoglu, 2012), and reduce their psychological pressure by enabling them to give remote feedback asynchronously (Ho and Savignon, 2007). However, positive outcomes and advantages of online PF are not assured by these study findings. Contrary research also uncovered drawbacks of online PF. Guardado and Shi (2007) reported that ESL university students often ignore a significant amount of online PF because they find it more difficult to clarify and negotiate meanings online than in person. Online PF, according to Cheng (2009), did not make the student more motivated, engaged, or autonomous writers. The mixed findings about online PF’s effects have highlighted the significance in continuously exploring how online PF can be effectively managed and incorporated into writing instruction. Research on how different modes of online PF can be appropriately combined and magnify their benefits for L2 learners’ writing development is therefore highly required (Yu and Lee, 2016).

### Technological features of online peer feedback

Previous studies usually identified computer-mediated PF as either synchronous or asynchronous depending on the types of computer-mediated communication (Yu and Lee, 2016). As stated earlier, the emerging online tools have reformed today’s language learning paradigm and challenged the established categories of the online learning context (Kessler, 2018). Innovative online tools have offered opportunities for language learners to study across synchronous and asynchronous settings, interact and collaborate extensively and intensively through authentic learning materials in a variety of modalities (e.g., text, audio, video, and images). The unique affordances of each online tool can support learners to realize different aspects of learning purposes and objectives and bring new forms to PF activities [for a review, see Chen (2016)]. Thus, instead of simply classifying online PF as synchronous or asynchronous, we attach more importance to technological tools’ ever-changing affordances and features, and sort out online PF based on mediating tools’ unique functions and advantages.

#### Moodle feedback

The extensively used LMS platform, Moodle, has given students access to a complete learning environment that supports them in all aspects of learning, including course preparation, requirements, learning instruction, comments, discussion, and evaluation (Beatty Connie, 2006; Hamid et al., 2014; Irwin, 2019; Fernando, 2020). The PF handled on Moodle has displayed its own features. For example, Moodle effectively helped students give formative feedback in academic writing with clear feedback rubrics from teachers (Fernando, 2020), especially after students were given adequate training (Irwin, 2019). PF content from Moodle is formal, targeted, and concrete (Nelson and Schunn, 2007; Fernando, 2020; Liu et al., 2021). Moreover, the Moodle forum generally serves the correspondent teacher for evaluation (Beatty Connie, 2006), guidance, and supervision (Irwin, 2019), while students regard giving feedback on Moodle as a kind of responsibility or assignment (Beatty Connie, 2006; Liu et al., 2021). As Moodle was designed exclusively for educational purposes, students tended to give feedback by following instructions thoughtfully and carefully.

#### WeChat feedback

A noteworthy research line is the use of social media for PF in writing. PF mediated by social media such as Facebook (Petrovic et al., 2014; Demirbilek, 2015; Lin, 2018), blogs (Nguyen, 2012; Zhang et al., 2014; Chen, 2016), Instagram (Nushi and Dayani, 2022), WeChat (Xu and Peng, 2017; Ma, 2018) and Wiki (Irwin, 2019) were given in more extensive and diversified forms. Most previous research primarily identified the advantage of easy connection and message transmission between teachers and students on social media (e.g., Petrovic et al., 2014; Demirbilek, 2015; Xu and Peng, 2017; Lin, 2018). According to Demirbilek (2015), students found Facebook easier to use mainly because of its pervasiveness and users’ familiarity with the tool’s functions and interface. Students also highlighted the strong interactive features of Facebook, which enabled them to stay connected with peers whenever needed. Similar findings were obtained by studies using WeChat as a feedback delivery tool (Xu and Peng, 2017; Ma, 2018). Xu and Peng (2017) showed that Chinese L2 learners also favored using WeChat due to its ease of use and ubiquitous access. Ma (2018) found that university L2 learners praised WeChat feedback for its success in turning the English writing course into an interactive process. However, limitations of social media-mediated PF were also identified. For instance, PF given through Facebook was criticized for being too vague, unclear, and less helpful for feedback receivers to revise their writing (Demirbilek, 2015). The content of WeChat feedback was found superficial and seldomly addressed in-depth problems (Ma, 2018). The interaction on social media may distract students from learning as it was not originally designed for educational purposes (Petrovic et al., 2014; Ma, 2018). Additionally, non-anonymous interaction on social media would cause potential barriers for learners to give critical, negative, but informative feedback (Lin, 2018).

#### Rain classroom feedback

Professional teaching tools that are simple to use and offer more strong interactive functions have emerged as a result of the rapid growth of Web 2.0 technology. Rain Classroom is such a specialized tool. The primary function of Rain Classroom is to project the teacher’s (i.e., the presenter) lecture slides to the audience’s mobile application. Rain Classroom differs from other online tools in supporting real-time feedback on the spot. That is, the audience can comment on the presenter’s speech and synchronously send their feedback to the presenter’s computer while the presentation is ongoing. The contents of the synchronous feedback can be chosen to display on the classroom screen by the presenter, either anonymous or identified. To date, the real-time feedback on Rain Classroom has drawn researchers’ attention. For instance, previous studies found that the application of the Rain classroom enabled learners to give their responses immediately as soon as they were required to do so (Sun, 2019). Rain classroom provided students with a platform for giving timely feedback as soon as they spotted any mistake in format, language, content, or structure (Yu and Yi, 2020). The synchronous characteristic of Rain Classroom-based activities ensures that learners play a central role in the learning activity (Xiangming and Song, 2018). In addition, the Rain classroom promoted students’ collaboration and helped them to acquire a sense of ownership in their learning (Yu and Yi, 2020). Nevertheless, since Rain Classroom is a new tool that has recently been used in teaching, it is unclear whether and how it will support academic writing, which, therefore, deserves to be explored in our study.

### Learners’ social-affective dispositions toward online peer feedback

From the perspective of collaborative learning theory in L2 acquisition, PF contributed to linguistic knowledge acquisition and writing skills developing mainly through facilitating social support and scaffolding among peers in a socio-interactive environment (Yu and Lee, 2016). As it takes place in the socio-interactive context, PF is regarded as a social practice in which interpersonal relationship management serves as a source of emotions that impact their study (Yang and Carless, 2013). During a PF activity, students must be cognitively, behaviorally, and affectively engaged (Zhang, 1995; Cheng and Hou, 2015; Zhou et al., 2020). That is, in addition to focusing on the feedback contents, feedback participants would experience positive (e.g., satisfaction, pride, and trust) or negative (e.g., anger, anxiety, and embarrassment) feelings aroused by giving and receiving feedback, and the feelings in turn influence their interpersonal relationships, accepting feedback, and positive learning experiences (Yang and Carless, 2013). Negative feelings would threaten students’ sense of identity and self-esteem (Crossman, 2007), and prevent them from help-seeking (Liu et al., 2021), while positive feelings could foster trust, empathy, and support among peers (Värlander, 2008). Briefly stated, learners’ affective dispositions toward PF are of great importance because they cannot only influence the success and effects of PF activity, but also closely relate to other important psychological elements such as learning motivations, self-esteem, and stance (Värlander, 2008; Yu and Lee, 2016).

Given that PF is a social practice where learners need to manage a source of emotions, Yang and Carless (2013) have conceptualized a feedback triangle framework with a *social-affective* dimension. The social-affect dimension mainly attends to the social and interpersonal negotiation of feedback, how information about the learners’ social roles in the learning settings is transferred by feedback, and how their emotions are engaged throughout the feedback activity. Based on their research, Zhou et al. (2020) proposed five key components, including feedback content, feedback design, situational context, individual differences, and mutual respect that can influence students’ social affect in the feedback process. They emphasized peer trust and respect as two of the five influencing elements, arguing that lacking mutual respect may prevent the student from feeling satisfied with PF. They also urged that future studies pay more attention to students’ social-affective dispositions, which permeate PF process and influence the potential learning objectives.

While it is widely recognized that integrating online tools boosts students’ learning effectiveness in feedback activities (Cunningham, 2019), researchers have also shown a growing interest in exploring learners’ affective dispositions in online learning. Despite the mixed findings in the extant literature, several previous studies have laid a basis for us to further inquire into learners’ social affective dispositions toward online PF. For example, in a distant class, Sun et al. (2019) highlighted the role of emojis in increasing peers’ awareness of their affective status, and establishing a general informality of PF activity. Liu et al. (2021) also suggested that emoji functions of online platforms could soften the communication atmosphere and help peers to express their true feelings. In addition, based on the anonymous function of Facebook, Lin (2018) found that non-anonymous participants were likely to give more affective feedback, showing opinions as supporting or opposing, while anonymous participants gave more cognitive feedback. According to a research synthesis by Chen (2016), online feedback reduced students’ worry about face-threatening problems, non-native accents and bias caused by social norm, and the absence of face-to-face communication benefited students with different cultural backgrounds who appeared to be less active in classroom interactions. However, on the contrary, Chen (2016) reminded us that lacking verbal communication in online feedback might cause learners’ negative attitudes toward learning.

We draw the conclusion from the reviewed findings that the integration of technological tools has created new scenarios and established new norms for students to engage in PF. Learners seem more sensitive to their social roles and interpersonal relationships while interacting with their peers through technological tools. Although previous studies suggested that technological tools can provide new ways for learners to deal with potential affective problems, how L2 learners would take advantage of the combined use of technological tools to handle social affect remains unknown. Taking the social-affective view on feedback (Yang and Carless, 2013), we would primarily target the social-affective aspects of PF process concerning L2 learners’ social roles and peer interactions.

### The current study

With the above-analyzed unique features of Moodle, WeChat, and Rain Classroom feedback, L2 learners’ perceived advantages and social-affective disposition are pinpointed as research foci in the present study. Specifically, we aimed to explore how the combined use of Moodle (offering a complete overview and guidelines of the course), WeChat (attending to participants’ affective factors), and Rain Classroom (providing on-the-spot feedback) can work more effectively for delivering PF in an academic writing course based on the experiences of 12 doctoral students. Combining the reviewed literature, we intend to answer the following two research questions.

(1) What are the students’ perceived advantages and disadvantages of online PF (i.e., Moodle, WeChat, and Rain Classroom feedback) in terms of their technological features?
(2) How do the technological features of online PF affect students’ social-affective dispositions during the PF activity?

## Materials and methods

### Action research design

**FIGURE 1:**
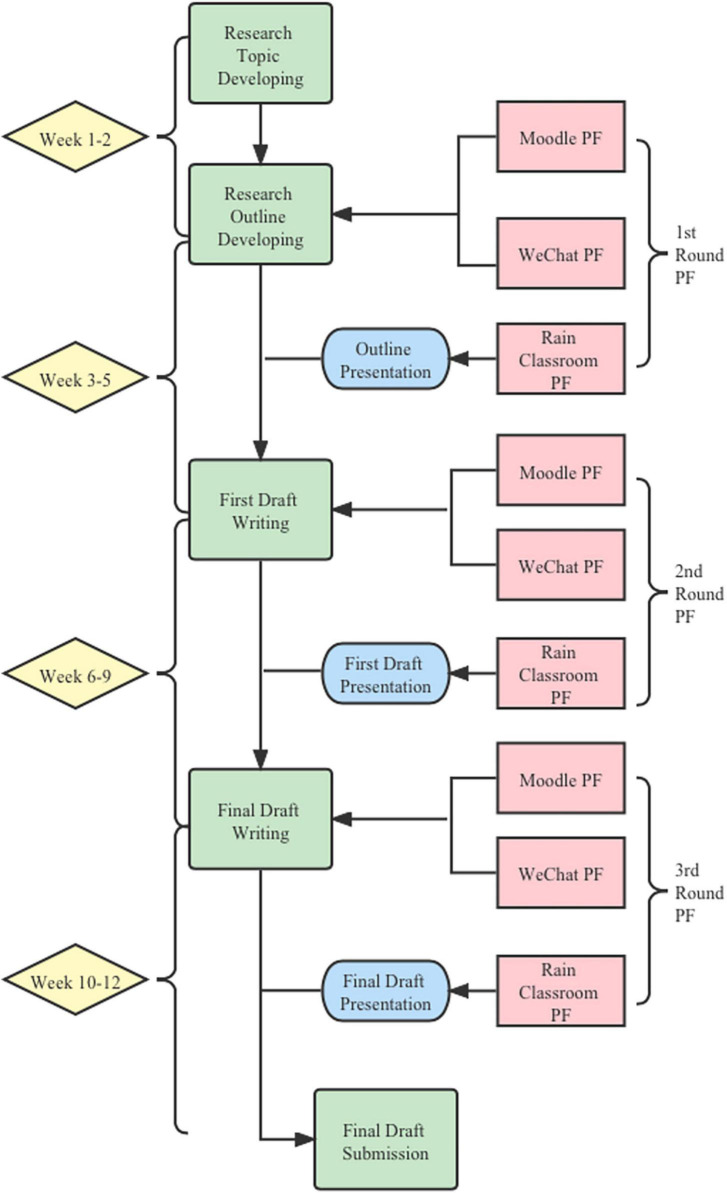
The course plan.

This present study incorporates a spiral of actions (Kemmis et al., 2014): planning, acting, observing, reflecting, and revising with obtaining evidence to better understand or enhance the aspect of PF. The teacher of the writing course, also the principal investigator of this research project, had a long-term reconnaissance on PF in the previous course before planning and conducting the current research. In order to optimize the outcome of PF activity, the teacher formulated a 12-week teaching plan facilitated by three technological tools (see Figure 1), and then put it into action, collected and analyzed data to see the effects of the plan. Guided by practical action research methods, this study involves “the use of qualitative, interpretive modes of enquiry and data collection by teachers with a view to teachers making judgments about how to improve their own practices” (Kemmis et al., 2014, p. 11).

### Participants

The research participants were 12 doctoral students taking up the academic writing course. They were from one intact class. As shown in Table 1, their age ranged from 24 to 41 years, with a mean age of 29.6. Four of the participants were male and eight were female. Two of the students were from Macau, a special administrative region of China, and the remaining 10 were from different provinces of China. The participants’ major backgrounds varied, which mainly included education, psychology, and business administration. To protect participants’ identities, pseudonyms were given to participants while reporting the results (see Table 1). The teacher had reached consent with the participants on data collection at the beginning of the course, and participants were allowed to quit the study at any time.

**TABLE 1:** Participants’ information.

Name	Gender	Age	Province	Educational backgrounds
Melody	Female	29	Sichuan	Business English & Education
Katy	Female	24	Macau	Educational Psychology
Skyler	Male	25	Guangdong	Physics Teaching
Stella	Female	35	Tianjin	Science Education
Apple	Female	26	Anhui	Preschool Education
Jessica	Female	25	Shanxi	Physical Education
Jay	Male	31	Hunan	TESOL
Lily	Female	25	Shandong	English Teaching
Sherry	Female	30	Beijing	Literature and Art
Bobbie	Female	41	Guangdong	Marketing Management
Joseph	Male	30	Jiangxi	Pedagogy
Bill	Male	34	Macau	Business Administration

### Course plan

#### The aim of the course

This action research was conducted in an academic writing course setting at a public university in Macau, China. This course aimed to provide first-year doctoral students with introductory knowledge and hone their skills for educational research proposal writing. At the end of the course, students were expected to be able to understand professional terms, define key concepts, clarify basic writing steps, evaluate, and apply writing strategies relating to an educational research proposal.

#### Learning activities

**FIGURE 2:**
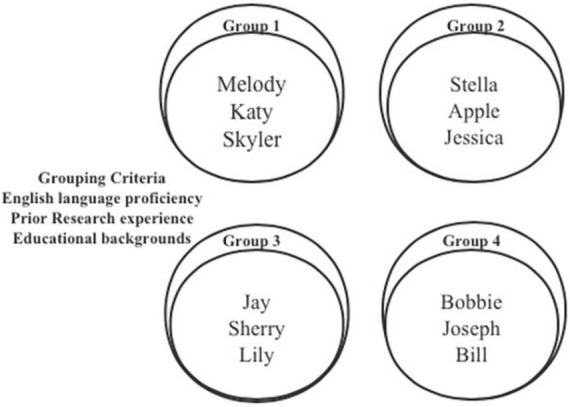
The learning groups.

The course was delivered mainly in the form of seminars, with three hours of teaching per week for 12 weeks. A variety of learning activities, including lectures, hands-on exercises, face-to-face, online feedback, and presentations, were taking place across in-and-out of class. At the beginning of the course, the teacher assigned the students an academic background survey to obtain information on students’ self-reported English proficiency and the number of academic publications. The teacher divided the 12 students into four groups to take advantage of group learning (see Figure 2). The grouping criteria were based on the survey results. The teacher considered the learners’ language competency, research experience, and educational backgrounds to balance the group capacity. Group members were given opportunities to share each other’s writing tasks, give, and receive PF within the group.

#### Schedules of the course

This action research primarily intended to enhance the effectiveness of PF for academic writing, PF and process writing pedagogies were jointly used during the course. As shown in Figure 1, the course plan comprised four major writing sessions: research topic outline development, the first and the final draft writing. Each writing session lasted about two to three weeks. Students needed to complete the required writing and feedback tasks throughout each session. For example, students were required to create subjects of interest and compose a research outline within the first two weeks. Once they completed their research outline, they would share their research outline and seek feedback from their group members *via* Moodle group forum and WeChat group chat. Before entering the next writing session, the teacher would organize a classroom presentation for students to report their drafts and gather real-time feedback from the whole class through Rain Classroom. After the four writing sessions, the students were expected to submit the final version of the writing work at the end of the course.

### Integration of technological tools

The teacher intentionally selected three technological tools to deliver PF by synthesizing their featured functions and affordances. Specifically, Moodle was used to deliver formal PF relating to writing skills and contents; WeChat was employed to conduct informal PF, allowing students to elaborate, reflect and discuss the feedback more freely; Rain Classroom was incorporated to collect real-time PF on students’ classroom presentations.

**Moodle** assisted the teacher in managing the whole course by sharing course materials, supporting the online forum, collecting, and grading students’ writing assignments. More crucially, the online forum on Moodle was used as a formal way for conducting online PF within the groups. In the first week of the course, the teacher set up four online forums in which the group members commented on one another’s writing drafts formally. The teacher had uploaded a list of prompting questions (see Table 2) as the criteria for students giving PF. By following the prompting questions, the students provided Moodle feedback on counts of research skills (e.g., topic design, introduction, literature review, etc.) and English language skills (e.g., accuracy, clarity, conciseness of writing, etc.). The teacher also made Moodle feedback a must-do task, requiring the students to use their real names when posting feedback, and checked the contents of Moodle feedback consistently.

**TABLE 2:** Teacher’s question prompts for research outline writing.

Sections	Question prompts
Introduction, Research Problems, Objectives, and Justification	1. What topic is studied? What aspects of the subject are studied? 2. What problem do you want to address and is this research project in response to this problem? How do you know? How can you determine topic? 3. What specific research questions should be asked in order to find out the gap/discrepancy between the optimal and the actual situations in the following areas: cognition, motivation, self-regulated learning, etc.?
Literature Review, Significance of Study	4. What does prior literature say about your topic? What and why is it of theoretical and/or practical significance from the previous related research or investigated literature? 5. How do your own research questions relate to the significance of your study? How might your research make a contribution to the area?
Methodology	6. How is the study conducted? What main research techniques (survey, interviews, case studies, modelling, etc.) you might use? 7. How are you going to collect the data? What data collection procedures are you going to follow? What are the data sources? Are there any possible difficulties for data collection?

**WeChat**, a social media application, was also selected as one of the tools to deliver online PF. WeChat has been immensely popular in China over the recent decade for its ease to use, multiple and practical functions (e.g., sending text and audio messages, emojis, memes, attachments, making audio and video calls, etc.). In addition, Moments, the social function of WeChat, allows its users to post texts and photos, share articles or music with their WeChat friends. Their WeChat friends can then give them “a comment” or “a like” to the newly posted content. Due to its entertaining features, social media used in PF could strengthen the feedback activity’s interactivity and the student’s engagement. Thus, the group members were required to join a group chatroom on WeChat during the first week of the course, through which they freely shared thoughts and reflections for three rounds while they were working on the three writing tasks.

**Rain classroom^1^** is an online teaching toolkit connecting PowerPoint and WeChat. The main function of Rain Classroom is to project the presenter’s slides to the audience’s WeChat mobile application. It also allows real-time interactions such as sending instant feedback, voting, raising, and answering questions between the presenter and the audience. The teacher took advantage of Rain Classroom’s slides-sharing and real-time interaction functions to facilitate the three-round writing drafts presentation. That was, with projecting the student presenters’ slides to their peers’ mobile phones, each of their peers should give real-time feedback to the presenters while the classroom presentation was ongoing. Once the presentation was completed, the teacher would display all the anonymous real-time feedback on the classroom screen.

### Course implementation

The academic writing course began in September and ended in December 2018. By and large, the course was enacted as the original course plan: the students completed topic selection by Week 2 and presented the research outline to their peers by Week 6. They then started to write and present the first draft by Week 9, and completed their final draft within the last 3°weeks. From Week 2 to Week 12, the three rounds of PF respectively through Moodle, WeChat, and Rain Classroom for commenting outline, first and the final draft had also been done.

The teacher and two research assistants participated in the whole action process: the teacher collected data such as writing assignments and PF, and the two research assistants made classroom observations, took field notes, and interviewed students three times when each round of PF finished. Combining the flaws perceived and reported by several students in the interviews as well as the teacher’s own reflection, the teacher adjusted the original course plan at Week 11 by adding a face-to-face PF session to the course on Week 12.

### Data collection

According to Elliott (1976), the data for action research should be gathered from multiple perspectives. There should be at least three different perspectives respectively from the teacher, the students and the observers. Thus, this study had collected multi-perspective data including: (a) PF emerged on technological tools and those generated during the face-to-face session on Week 12, (b) student interviews, (c) students’ writing assignments, (d) researchers’ field notes.

#### Peer feedback

There were two types of PF data collected during the course. The first type, online textual feedback was generated and exported from Moodle, WeChat, and Rain Classroom. The other type, face-to-face verbal feedback, was recorded by the two research assistants in the face-to-face feedback session.

#### Interviews

Semi-structured interviews were conducted and recorded by the two research assistants three times when each writing task was completed. Students were asked about their perceptions of the usefulness of PF and their emotional reactions to the PF. Examples of interview questions were: How did you feel about using Moodle/WeChat/Rain Classroom to give online PF? How do you compare the usefulness of online PF giving and receiving through Moodle, WeChat, and Rain Classroom? Do you think that PF helps you improve your writing skills or performance and how, please give examples? Did you go through any positive or negative emotions when you participated in PF activities, please give examples?

#### Students’ writing assignments

The 12 students’ writing assignments, including the research outline, the first and final wiring draft were all required to be submitted through Moodle. There was a total of 36 writing assignments collected by the teacher at the end of the course.

#### Researchers’ field notes

Two research assistants observed the whole course. While they observed the course implementation, they took field notes regarding the in-class learning activities, engagement, performance, and interactions among the teacher and students.

### Data analysis

Given that the study was action research, we were simultaneously collecting and analyzing data as the study proceeded. Such was done because it allowed the researchers to reflect on and adjust the ongoing research actions continuously. Based on Miles and Huberman (1984) qualitative data analysis methods, the data analysis process consisted of four main steps: reading all the data carefully, selecting relevant data, presenting the data, and interpreting and drawing conclusions.

We first verbatim transcribed all non-textual qualitative data, namely the audio recordings of interviews, and face-to-face feedback. And then, the transcripts along with other textual materials (i.e., online PF, writing assignments, and researchers’ field notes), were imported into a qualitative software, NVivo 11. All researchers carefully read through all data and made an initial selection: Data that had little relevance to the topic of PF (e.g., comments on the teacher’s teaching style; suggestions for improving teaching design) was discarded in this step. Using NVivo’s cross-text reading feature, all researchers together created a first-order code of the data based on the content of the data, the research questions and interview protocols. By examining the first-order codes once again, three researchers independently grouped similar meanings into the same code, merged repetitive codes, and then built a new second-order code. After the independent work, they discussed inconsistent parts to reduce recurring codes, cross-checked references, and finally agreed to categorize the second-order code into three final themes.

In addition, the triangulation strategy was applied to safeguard the whole data analysis by comparing data from multiple sources (Creswell and Poth, 2016; Yin, 2017). For example, the results of students’ interviews were corroborated by researchers’ field notes and students’ writing assignments. Additionally, the member checking strategy (Bernard et al., 2016; Creswell and Poth, 2016) was also utilized, which entailed discussing findings with participants to seek their comments.

## Results

### Moodle feedback, addressing research-related issues of academic writing

Overall, students considered online PF useful for revising writing work. And the more useful they perceived, the stronger willingness they would have to give online PF. This finding was firstly evidenced by the ever-growing number of PF throughout the whole feedback activity. As shown in Table 3, the quantity of online PF was consistently increasing over time. The quantity of WeChat feedback increased most rapidly, while the number of Moodle and Rain Classroom feedback grew relatively slowly. However, consistent growth has been displayed on all three platforms. The growing trends indicated that the students kept contributing more feedback to their peers in the three rounds of PF activity. Furthermore, each of the three technological tools had its own features and advantages that promoted online PF in different ways.

**TABLE 3:** Number of peer feedback collected.

Types of feedback	Writing task	Number of feedback collected	Total number of feedback collected
Moodle	Research Outline	31	136
	First Proposal Draft	43	
	Final Proposal Draft	62	
WeChat	Research Outline	142	513
	First Proposal Draft	154	
	Final Proposal Draft	217	
Rain Classroom	Research Outline	65	244
	First Proposal Draft	82	
	Final Proposal Draft	97	
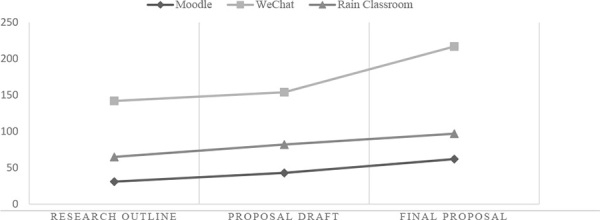			

We have classified online feedback into two major types based on its contents. One pertained to research skills such as the research topic selecting, literature searching and reviewing, and educational methods conducting. At the same time, the other focused on English language use, such as word choice, grammar, sentence structure, meaning comprehension, format, and style. By counting the number and coding the contents of Moodle feedback, we found over 80% (25 out of 31) of the Moodle feedback for research outline writing, near to 70% (30 out of 43) for first proposal draft writing, and about 65% (40 out of 62) for final proposal draft writing belonged to the category of research-related issues. It showed that Moodle feedback mainly attended to research-related issues of academic writing. Additionally, we have recorded students’ explanations for their inclination to provide more research-related feedback on Moodle.

Moodle, as an online learning management system, allowed the course administrator (i.e., the teacher) to play an instructional role in Moodle feedback activity. Hence, the teacher uploaded a list of question prompts (see Table 2) to scaffold students in drafting a research outline in the first week of the course. As the questions raised by the teacher mainly focused on research skills and strategies, the students were likely to imitate the model and take it as the criterion for evaluating each other’s drafts. Without much prior knowledge of doing research, some participants articulated that it was safe to ask peers similar questions as the teacher did.

From the standing point of feedback givers, teachers’ scaffolding materials on Moodle benefited their formulation of PF in the manner of educational research. For example, Melody shared her view on the teachers’ guidance on Moodle, *“Having no research experiences before, I just did not know how to start giving PF. It was the teacher’s Moodle guidance that let me know the right way.”* Another student, Skyler, who rated himself the lowest score for English proficiency, also highlighted the value of guiding materials on Moodle when he gave other kinds of online PF, *“Almost whenever I posted Rain Classroom feedback, I would refer to Moodle guiding materials. It was a reference for me to give right feedback on educational research.”*

From the standing point of feedback receivers, several students also confirmed the usefulness of Moodle feedback in addressing research-related problems. As Jessica commented,

*“The teacher’s prompting questions on Moodle assist us to construct the research proposal step by step. Following the prompting questions, my group peers can discuss and collaborate within a well-structured framework. PF following the prompting questions on Moodle kept us staying on the right track of doing research.”*

### WeChat feedback, clarifying and extending moodle, and rain classroom feedback

Compared with Moodle feedback, WeChat feedback was more about clarifying, elaborating, reflecting, or developing the contents of feedback received through Moodle and/or Rain Classroom. After fully explaining on WeChat, Moodle, and Rain Classroom feedback would be considered clearer and more informative for revising their writing work. We compared the revised contents of students’ writing drafts to see whether the students had accepted the online PF and what types of feedback had been adopted. The comparisons were made by examining each draft and marking the major revisions first. And then, the contents of PF were examined and matched with the revisions if possible. As shown by the comparison results, feedback put forward on Moodle and/or Rain Classroom, and then discussed on WeChat was most likely to be taken up by the students. Interviews with three students, Melody, Sherry, and Bobbie corroborated this point by revealing that the most useful feedback they had received was from WeChat. Melody and Bobbie also provided reasons for why they tended to adopt such feedback.

*Previously, I got two feedbacks from Moodle which advised me to revise my theoretical framework. I also felt that the theoretical framework was the weakest part but I struggled to find effective solutions. However, I did not start revising it until Skyler provided me with some very useful references on WeChat…WeChat feedback is a wonderful extension to Moodle and Rain Classroom feedback.* (Melody)

*I like listening to my peers’ feedback on WeChat because I like how they elaborate on their feedback. Besides, unlike Rain Classroom, WeChat allowed me to give them my responses to their feedback conveniently. WeChat turned their feedback to be more convincing.* (Bobbie)

Owing to its strong interactive feature and ubiquitous access, WeChat feedback has played a significant role in supplementing the contents of Moodle and Rain Classroom feedback. Despite Moodle and Rain Classroom feedback being good at pinpointing wiring issues directly, considering the complexity of academic writing, further explanations to the feedback were still needed to some extent.

### Rain classroom feedback, identifying surface-level mistakes effectively

Meanwhile, the real-time feedback on Rain Classroom, which was not available on Moodle or WeChat, proved advantageous in tackling surface-level mistakes. In this study, surface-level mistakes were recognized as easily identified mistakes that need no further examination or argumentation. This category of problems mainly reflected the readers’ initial impression and confusing points about the drafts. It included basic language errors such as spelling, grammatical, inappropriate collocations, and basic research-related problems such as inaccurate reference style, misconceptions of research methods, violation of research ethics, etc.

Most Rain Classroom feedback was found to be effective in detecting such surface-level mistakes. For instance, typical Rain Classroom feedback read “*Too brief on data analysis*” (Sherry), “*The literature cited in the introduction section was too out-of-date*” (Skyler), and “*There was something wrong with the APA Style.*” (Jay)

Although it was short in length and not fully developed in contents, Rain Classroom feedback was considered helpful in informing the feedback receivers of minor mistakes in their writing. Unlike in-depth questions that require repeated thinking, the surface-level questions were easily identified but often ignored by the author himself or herself for various reasons. The Rain Classroom provided an opportunity for peers to find out the primary errors and authors to address them promptly. *“Although feedback messages from Rain Classroom were often concise, they did help me address the small mistakes in a short time*,” shared Jessica.

One researcher recorded how the students used Rain Classroom to give real-time feedback in class,

“*According to my classroom observation, the students showed interest in sending real-time feedback via Rain Classroom on their smartphones. When the teacher displayed PF on the projection screen, some students would nod their heads and take notes. Students were actively engaged in typing their feedback on their smartphones and several students even stayed in the classroom to discuss the Rain Classroom feedback with their peers after class. Rain Classroom was like a precursor who helped the students clear away the primary barriers before they penetrated their writing.”*

### Rain classroom feedback, lacking in-depth interaction

Students also reported perceived drawbacks along with the observed effects and advantages of online PF. Of the three types of online PF, complaints concerning Rain Classroom’s lack of in-depth interaction and rising cognitive load had been made the most frequently.

The contents of the real-time feedback on Rain Classroom were shown to be brief and contained little in-depth interaction. As mentioned above, often students merely remained on the surface-level problems without further probing. According to the interview data, although Rain Classroom feedback was helpful for them to address the basic mistakes, due to its requirement of synchronously typing on the mobile phones, it restricted them from digging deeper into the problem. Jay pointed out that *“The Rain Classroom feedback is limited in length. It is impossible to deeply communicate with each other by typing in such a short time.*” They also reported that the use of Rain Classroom had increased their cognitive loads and distracted their attention from their peers’ presentations. As Joseph commented, “*As we were typing our ideas on mobile phone while the presentation was ongoing, we would certainly miss some contents of the presentation*. *Rain Classroom highly demands multitasking abilities which I am lacking.*” Katy explained the conflicts she perceived between using Rain Classroom and academic writing.

*Unlike any other kind of writing, academic writing usually requires critical thinking skills. Therefore, the feedback on academic writing needs further evaluation, argumentation, or reflection. However, since Rain Classroom feedback restricts deep thinking, it goes against the nature of academic writing.* (Katy)

Considering the students’ reflections on the flaws of Rain Classroom feedback, the teacher added a face-to-face session of feedback in the last week for them to address deep questions. This pedagogical adjustment was found effective based on the interview data and researchers’ field notes. Four students spoke highly of the complementary function of face-to-face feedback. For example, Bill noted, “*I still prefer to give face-to-face feedback on writing work, because more substantial information can be delivered, and I can argue back if I disagree with the feedback*.” Bobbie added, “*I think face-to-face feedback is irreplaceable since spoken language is much more efficient in elaborating and arguing ideas*.”

The researcher’s field notes also confirmed the usefulness of face-to-face feedback,

*“On the last day of the course, right after the final proposal presentation, the students immediately devoted themselves to the face-to-face discussion. They showed strong eagerness to communicate with one another, some talking and laughing, some discussing seriously. The atmosphere in the classroom was warm.”*

### Emojis and memes showing respect and care for peers’ feelings

According to data retrieved from student interviews, giving, and receiving online feedback would trigger varying sorts and degrees of emotions. Students were prone to experience unpleasant emotions like embarrassment, discouragement, or worry especially when giving and receiving feedback containing negative information. They believed that once such emotions occurred, the effectiveness of feedback activity would be largely curtailed. Most students articulated that they had consciously avoided evoking their peers’ unpleasant feelings in the delivery of online feedback. Moreover, WeChat was the most frequently mentioned tool to appropriately deal with their affect in PF process.

First and foremost, emojis and memes sent through WeChat can mitigate feedback receivers’ negative feelings brought by unfavorable feedback contents. We found a pattern that critical feedback contents were usually accompanied by emojis or memes showing smiley, cheer, pleasure, or salute. Melody verified this point in her interview, “*Each time I need to deliver harsh things to my peers, emoji and meme must be used together.”* Asked why she liked sending emojis and memes with critical feedback, she continued, “*For online feedback, the feedback receivers cannot see our faces or hear our tones, emojis, and meme will then represent our humble attitudes*.” Melody’s point of view was echoed by another student, Joseph, who had received critical WeChat feedbacks for several times.

“*To be honest, I do not feel like being criticized by my peers since it makes me feel awkward. We are all doctoral students, and nobody should win an upper hand over knowledge or profession. Thus, it will be hard for me to accept them if they do not show humbleness or sincerity when giving criticisms. Luckily, I get adequate respect from the attached emoji or meme, which let me feel much better and know that my peers must be very conscientious when commenting on my writings.”* (Joseph)

For the students, using emojis and memes in online feedback did not only directly soften the harsh feedback tones and strengthen politeness, but more importantly, it better demonstrated their humble attitudes, respect, and care for peers’ feelings about receiving negative feedback contents. Such respect and care foster more positive feelings like support, empathy, and trust.

### One-to-one conversation window reducing face-threatening problems

WeChat not only supported the use of emojis and memes, but it also provided appropriate feedback delivery scenarios that helped students deal with affective issues. In their interviews, several students often stressed “face”-threatening problems they had undergone in the PF process. “Faces,” in their own language, meant the need to protect self-esteem and identity in front of their peers. They thought receiving negative feedback on public occasions was especially easy to evoke strong unpleasant feelings of losing face, as negative comments were like a public announcement of lower writing proficiency. They explained it was not because they could not listen to peer criticisms but because the criticisms sent on public occasions would harm their professional images as Ph.D. students. Thus, they needed a “face”-maintaining way to receive and read negative feedback.

Luckily, the students found a private setting for delivering negative feedback through the online platform. On WeChat, students set up one-to-one conversation window as a “private setting.” Several students disclosed that they had used one-to-one conversations on WeChat for such a purpose. For instance, Bill recalled an example about how his classmate, Jay sent explanations to him using a one-to-one conversation window.

*“Once, Jay had made a few harsh comments on my presentation in the classroom. Although some of the comments targeted my problem, they still embarrassed me and made me lose face. After the classroom presentation, however, Jay sent a message to me on WeChat privately, a long message that explained why he thought I should revise my work in his way. I think his private message calmed me down, and I started considering his opinions.”*

This finding revealed doctoral students’ psychological need for maintaining their self-image and professional status in front of others. They would feel upset, embarrassed, or insecure when the publicly delivered PF harmed their faces. Thus, making negative PF privately sent and received through a one-to-one conversation window could reduce the emotional problems aroused by losing face.

### Moments, the social function of WeChat increasing emotional load

Emojis, memes, and the one-on-one conversation window are all used by students to help with their affect, but WeChat’s social function, Moments, was found to be somewhat detrimental for them to deal with affective issues. The students claimed that they were already burdened by the peer social interactions on WeChat Moments. When the social interactions were dissatisfying, it would reduce their trust in one another, negatively impacting their mood about giving feedback to others. Stella illustrated her dilemma about using WeChat.

*As we added each other as friends on WeChat, it was inevitable to see their updates in Moments. In order to maintain good interpersonal relationships with my group peers, I have to comment on or send “likes” to their posts. Of course, nobody forced us to do so, but when everyone does so, you will probably follow it. If you turn yourself into an outsider, you will also become an outsider in group work.* (Stella)

Lily and Apple agreed with Stella’s views and shared their unpleasant feelings when they found they were blocked from seeing the updates in Moments by one of their group members.

“*I felt uncomfortable about being blocked by my peer. I would even doubt whether I did anything that offended her. I don’t like this kind of feeling that obviously lowered my trust in my peer*”. (Lily)

*“After knowing she blocked me, I felt strange to send her WeChat feedback the next time.”* (Apple)

Although WeChat had been praised for its strong social function of connecting people conveniently, it was just the ubiquitous connection that resulted in their emotional burden. Students were now connected by social media regardless of time or place. Such a strong connection made them lose their personal space to some extent. In order to achieve better learning results, they had to consider more about how to manage their online social interaction with their peers. The additional devotion to maintaining interpersonal relationships went against their will and thus resulted in the feeling of tiredness and anxiety. In the social media-based learning activity, the potential negative effect of online interaction cannot be overlooked, as it has inevitably increased the pressure of social interactions and weakened their trust in one another.

## Discussion

This study presents two sets of findings, which are informed by the two research questions. For the first research question, we have identified the learners’ perceived advantages and benefits of the three modes of online PF. It is found that the combined use of online PF was overall useful for students revising academic writing work. This finding was evidenced by the fact that learners were willing to contribute an increasing number of online PF throughout the 12-week course. We also analyzed the unique features and advantages of each online PF to promote the effectiveness of PF activity. Specifically, Moodle forum provides more formal contexts for the students to give and receive online PF. Following the teacher’s guiding materials, learners tend to express ideas in a more research-oriented format than in the other two platforms. Meanwhile, chat-based feedback given *via* WeChat can bring further clarification, elaboration, and reflection on the feedback received from the other two platforms. Besides, Rain Classroom, which allows real-time feedback, seems to have a more decisive advantage in tackling surface-level mistakes than the other two used in the current study.

The identified advantages of the three platforms enable us to contemplate possible ways of combining and maximizing online PF’s potentialities for serving academic writing. Given that the creation of academic papers involves a series of tasks evolving from the basic to more advanced ones, the whole process of giving feedback is also a spiral. During the feedback activities, the students need to apply basic research skills such as correctly searching literature, using citations, format, and style of academic manuscript, as well as handle more complex cognitive tasks like making the contents logical, coherent, and persuasive. Therefore, different affordances of the multi-platform can help with feedback targeting different levels of writing problems. For instance, the real-time feedback of Rain Classroom is appropriate for students to detect and reduce basic errors promptly. Nevertheless, due to its limitations on length and timing, it is not suitable for addressing more in-depth and ill-structured issues. However, Moodle’s features can well-complement this shortcoming of Rain Classroom. As Moodle supports the teacher’s mentoring role, the students can provide complete feedback to each other with the teacher’s step-by-step guidance. Since the diversity of opinions may play a key role in improving a student’s writing, particularly at the doctoral level of study, more unrestricted discussion of ideas is also needed to increase the inspiration and outcome of academic writing outcome. Due to its robust interactivity, WeChat strongly promoted free discussions in the academic writing process. Compared with Rain Classroom and Moodle, WeChat offers the students a more flexible and relaxing environment to explain and ponder previously received feedback. That said, the combination of three technological tools altogether fosters the richness of feedback production. Before a technological tool containing all needed functions emerges, teachers are advised to integrate different technologies to augment feedback production.

Meanwhile, students’ perceived disadvantages and drawbacks of online PF have also been recognized. The major flaw of online PF was manifested in the use of on-the-spot Rain Classroom feedback. Rain Classroom feedback may divert students’ attention from the content of other classmates’ presentations. Synchronous use of Rain Classroom to give feedback and evaluate others’ presentations challenged the students’ multitasking capacities so that students with lower English language proficiency or weaker multitasking abilities would easily encounter distraction problems. It undermines students’ comprehension ability when listening to the presentation. As a result, real-time feedback of Rain Classroom may work better in surface-level problems and seems less effective in dealing with more thought-provoking issues. The previous finding suggested that verbal communication was useful in clarifying and negotiating meanings between feedback givers and receivers (Zhu and Carless, 2018), the last week’s face-to-face feedback session provided a chance for students to evaluate, argue about, and reflect on their thoughts orally. In addition, for students with lower multitasking capacities, face-to-face feedback sessions also gave them the time and space to seek missed information and refresh their thoughts, hence strengthening their confidence in using online feedback in the long run.

For the second research question, we have explored the influence of online PF on students’ social-affective dispositions in the academic writing classroom. From the social-affective perspective, we captured and described varying sorts of emotions and affect relating to the students’ social roles and peer interactions. Specifically, the students showed positive emotions when seeing their peers’ humble attitudes, experiencing mutual respect and care for their feelings. Peers’ good intention to protect their faces can also avoid the generation of negative emotions such as embarrassment, anger, or upset. However, the ubiquitous connection on the social tool increased their emotional burden and curtailed peer trust. Compared with previous findings (e.g., Zhou et al., 2020), our finding further revealed how technological features of online PF can be adapted to address students’ social affective needs during the PF process.

In total, we have spotted three main functions of WeChat: emojis and memes, one-to-one conversation window, and Moments associated with students’ social-affective responses to online PF. Data obtained from WeChat feedback reflect that emoji and memes were frequently used, either along with text or sent alone when the students needed to protect their peers’ feelings. Some participants believed that the use of emojis and memes generated more positive emotions through passing on their humbleness and sincerity to the feedback receivers, while it also diminished negative feelings caused by conflicts, misunderstanding, or compromise. It strengthens the interpersonal relationships among students, creating a more harmonious and pleasant peer learning experience. The result also confirmed a growing body of studies regarding emojis as an essential component of cyber language. More researchers encouraged the use of emojis in the online learning context since emojis are useful for transmitting learners’ speaking tones and manners, and explaining metaphors in online dialogues (Sun et al., 2019; Liu et al., 2021).

In addition, we discovered that the feedback delivery scenarios (i.e., public or private occasions to give feedback) would affect learners’ emotions and their acceptance of the feedback in an online context as well. This finding is consistent with pertinent findings obtained from traditional face-to-face contexts (e.g., Belschak and Den Hartog, 2009). The student’s psychological need to protect their face was out to preserve their self-esteem and professional image in front of others. Otherwise, they would feel losing face, a feeling of embarrassment or even anger when they were publicly criticized or commented on. Compared with traditional classroom settings, online platforms allow learners to more flexibly choose conversation scenarios, such as in group or private chats, either anonymously or non-anonymously. The diverse scenario settings can better cater to students’ personalized needs in group learning. The one-to-one conversation window on WeChat, used as a private occasion to deliver negative feedback, was a good example demonstrating how students took advantage of technological affordance to protect their self-esteem.

Lastly, notwithstanding WeChat’s overall effectiveness in fostering students’ positive emotions, the overuse of its social function increased students’ emotional burden and harmed peer trust. Social media-based peer activities have inevitably intensified students’ online interaction. Students must put forth extra effort to maintain healthy interpersonal ties. For instance, several participants reported that they had to comment on or send likes to their peers’ Moments entries. The omnipresent social media much more powerfully binds students regardless of time and location. As a result, the social media-based peer activities call for greater commitments to sustaining individuals’ virtual identities, images as well as connections on the network. However, just because it takes more time and effort to maintain social media interactions, students will generally feel fatigued and stressed about utilizing social media for learning. Unfortunately, such worry and anxiety would undermine peer trust and divert their focus from the learning activity *per se*.

## Implications for practice and future studies

This study suggests three practical implications on how educational practitioners should design online PF activities to support the teaching of academic writing. First, with a variety of functions and affordances, massive technological tools on the market today are available for teachers to assist peer learning. Teachers are advised to compare and select the more appropriate tools based on the varying teaching contents, different levels of difficulty, and interactivity of the learning tasks. For example, social media should be given priority to peer tasks that call for strong interactivity or a flexible learning environment. In comparison, LMS should be used more when teachers engage in peer learning activities or directly scaffold the learners. A combination of the right tools can enhance the overall effectiveness of the peer learning process as well as optimize the learning products.

Second, it advises that teachers be attentive to the dual power of social media in peer learning. On the one hand, teachers may consider supporting the use of emojis, memes, and personalized conversation windows in PF activity to satisfy learners’ need for handling social affect. Through teachers’ verbal encouragement or demonstrations, students unaware of social media’s effectiveness in academic contexts can change their preconceptions. Learners are encouraged to share their examples of how social media are well used and explicitly evaluate each other’s ways of using it, enhancing their positive perception of such behaviors and further promoting them in the future. On the other hand, teachers must be cautious about the potential harm to peer trust caused by the ubiquitous interaction on these social tools. Teachers are recommended to establish guidelines and limits between students’ learning and social interactions on social media.

Finally, considering the cognitive affordability of the students who are not good at using real-time feedback tools, teachers’ overly reliance on online feedback is not encouraged in teaching and learning practices. Compared with face-to-face feedback, online feedback has its own advantages and disadvantages. If not constrained by contexts (e.g., online courses), teachers are advised to keep exploring the effective ways to combine both face-to-face and TPF approaches and design PF activities that meet certain groups of students’ needs as well as the learning objectives.

Meanwhile, this study also sheds new light on future studies regarding two aspects. For one aspect, as a supplement to quantitative studies that aim to measure the effectiveness of learning activities, learners’ experiences and perceptions of using a particular technology or a combination of several technologies are important for teachers to understand why certain activities are (not) effective in learning. Such knowledge is the basis for researchers’ further revision of research design and the selection of suitable technology that can better cater to learners’ needs. For another, it increased theoretical knowledge of the social affective dimension of feedback as it expanded the understanding of L2 learners’ social affective dispositions to feedback in the technology-assisted language learning environment. For example, having realized that incorporating social media could increase the students’ anxiety and pressure of social interaction, future studies are advised to continuously explore the possible technologies or/and teaching strategies that positively relate to their emotions and motivations. Future studies could also include more relevant social-affective variables such as students’ favor for the use of technological tools, interest in the new technological features, and find out the associations among them, so that we could more comprehensively understand learners’ psychological mechanisms and patterns regarding their peer collaborations and interactions through technologies.

## Conclusion

This current action research explored 12 doctoral students’ experience of using multiple online platforms to give and receive PF in academic writing. Informed by the technological features of multiple technological tools (i.e., Moodle, WeChat, and Rain Classroom) and the theories of social affect, it revealed two major findings: (1) students’ perceived advantages and disadvantages of each online PF in terms of technological features; (2) the influence of online PF on students’ social-affective dispositions to PF activity. The findings can add to the knowledge of technology integration and learning emotions about academic writing at the postgraduate level. Practical implications are also provided for teachers by showing them the technological capacities of different technological tools and enriching their technical resource pools when they need to plan technology-mediated PF activities. However, any endeavor to understand individual experiences through a qualitative study lens runs the danger of limiting generalization. Thus, our study should be considered explorative rather than conclusive since it is contextualized and cannot simply be translated to other learning contexts or online platforms. To gain further insights into the interplay between individual and contextual factors, the inclusion of other ethnic groups of L2 learners, or learners of different educational levels and in a wider range of technological conditions is necessary in the future.

## Data availability statement

The raw data supporting the conclusions of this article will be made available by the authors, without undue reservation.

## Ethics statement

Ethical review and approval was not required for the study on human participants in accordance with the local legislation and institutional requirements. The patients/participants provided their written informed consent to participate in this study.

## Author contributions

MZ: conceptualization, methodology, data curation, and original and final draft preparation. QH: conceptualization, original, and final draft preparation. JD: conceptualization. FL and BH: data curation and original draft preparation. All authors contributed to the article and approved the submitted version.

## Footnotes

1
https://www.yuketang.cn/en
